# Rectal squamous cell carcinoma treated with neoadjuvant fluorouracil, oxaliplatin, cetuximab, and radiation: A case report of pathological complete response

**DOI:** 10.1097/MD.0000000000038627

**Published:** 2024-06-21

**Authors:** Yuta Fujise, Shoichi Hazama, Toshiyuki Fujii, Motoshige Inoue, Shotaro Takahashi, Kazuya Yoshida, Akihiko Ikeda, Hiroshi Hashiyada, Kembu Nakamoto, Aogu Yamashita, Keisuke Hino, Kiwamu Okita

**Affiliations:** a Department of Surgery, Gastroenterological Center, Shunan Memorial Hospital, Kudamatsu, Japan; b Department of Internal Medicine, Gastroenterological Center, Shunan Memorial Hospital, Kudamatsu, Japan; c Department of Radiology, Tokuyama Central Hospital, Shunan, Japan

**Keywords:** cetuximab, chemoradiation, rectal cancer, SOX, squamous cell carcinoma

## Abstract

**Rationale:**

Treatment strategies for rectal squamous cell carcinoma (rSCC) are yet to be established, given its rarity. Although squamous cell carcinoma has been reported to be highly sensitive to cetuximab and radiation, there is no report of combination therapy of cetuximab and radiation for rSCC. In this study, we firstly reported a case of rSCC in which a complete response was achieved with the original chemoradiotherapy comprising oxaliplatin, S-1, cetuximab, and simultaneous radiation.

**Patient concerns:**

A 46-year-old women presented to our hospital with lower abdominal pain and fatigue.

**Diagnoses:**

Based on tumor marker analyses, histological examination of biopsy specimens, and comprehensive imaging, the patient was diagnosed with rSCC.

**Interventions:**

Neoadjuvant chemoradiotherapy (50.4 Gy) was administered in 28 fractions, along with concurrent chemotherapy comprising SOX (S-1: 80 mg/m^2^, days 1–5 and 8–12, oxaliplatin: 85 mg/m^2^, day 1) and cetuximab (400 mg/m^2^, day 1, 250 mg/m^2^, after day 8).

**Outcomes:**

Five weeks after chemoradiation, the patient underwent laparoscopic partial intersphincteric resection, achieving a complete pathological response.

**Lessons:**

This case firstly highlights the usefulness of SOX plus cetuximab combined with radiation in the treatment of locally advanced rSCC. However, a large-scale study is required to establish safe and effective treatment regimens.

## 1. Introduction

Colorectal cancer is the third most frequently diagnosed cancer worldwide and the fourth leading cause of cancer-related deaths globally.^[1]^ The majority of primary malignancies of the colon are adenocarcinomas. Primary colorectal squamous cell carcinoma (SCC) is an extremely rare subtype of colorectal cancer, with an incidence of <1% of all colorectal malignancies.^[2]^ Owing to the low incidence of this cancer and the lack of available literature, the underlying pathogenesis and risk factors are yet to be comprehensively confirmed.^[3]^ Moreover, a suitable chemotherapy regimen for primary SCC of the colon remains elusive.

In this study, we first report a case of rectal SCC (rSCC) in which a complete response was achieved with original chemoradiotherapy (CRT) comprising oxaliplatin, S-1, cetuximab, and simultaneous radiation and present a review of the literature.

## 2. Case presentation

**Figure 1.**
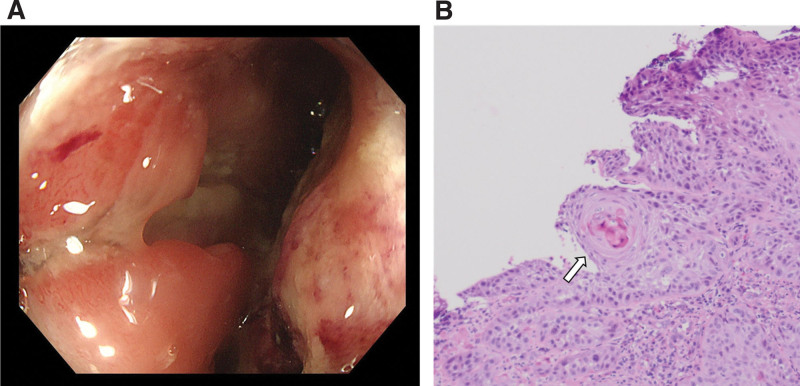
Endoscopy and pathological findings of rectal cancer. (A) Findings of lower gastrointestinal endoscopy. A circumferential tumor with stenosis can be observed in the lower rectum. (B) Pathology shows nested squamous cell carcinoma accompanied by cancer pearls.

**Figure 2.**
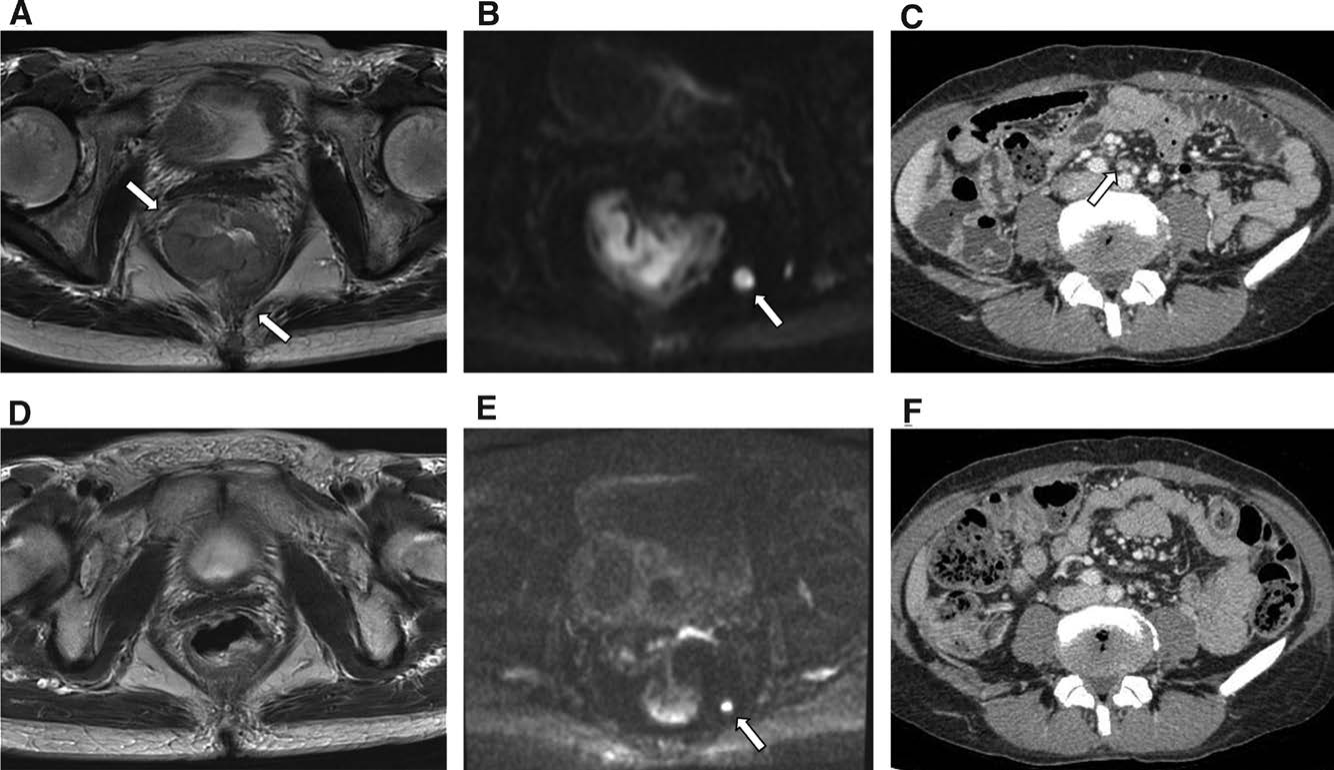
Findings of abdominal MRI and CT. (A) Abdominal contrast MRI shows the presence of a large tumor in the lower rectum. White arrows indicate the suspected extramural invasion. (B) Abdominal contrast MRI shows the presence of a round-shaped enlarged lymph node more than 10 mm in diameter at the pararectal region (white arrow). (C) Abdominal contrast CT indicated a lymph node 8 mm in diameter at the superior rectal arterial region (white arrow). (D) Abdominal contrast MRI indicating apparent tumor shrinkage. (E) Abdominal contrast MRI indicating apparent lymph node shrinkage (white arrow). (F) Abdominal contrast CT indicating that the previously seen enlarged lymph node has disappeared. CT = computerized tomography, MRI = magnetic resonance imaging.

A 46-year-old female patient presented at our hospital with lower abdominal pain and fatigue. The patient had no relevant medical history. Blood samples revealed elevated inflammatory response, with a white blood cell count of 15,700/μL and a C-reactive protein level of 1.15 mg/dL. Tumor marker levels were elevated, with levels of SCC-related antigen and cytokeratin 19 fragments at 4.1 and 5.0 ng/mL, respectively. However, levels of carcinoembryonic antigen and carbohydrate antigen 19-9 were within the normal range at 0.8 and 8.4 ng/mL, respectively. Colonoscopy revealed a circumferential type 2 lesion in the lower rectum (Fig. 1A). Histological analysis of biopsy specimens obtained at the time of colonoscopy revealed SCC (Fig. 1B). Abdominal magnetic resonance imaging (MRI) and computed tomography revealed a large mass in the lower rectum (58 × 41 × 78 mm). A break in the continuity of the rectal wall was observed, and extramural invasion was suspected (Fig. 2A). Numerous enlarged lymph nodes were detected around the locoregional nodes (Fig. 2B), including the superior rectal nodes (central nodes; Fig. 2C). The tumor was staged as T3, N2, M0, stage III C using the TNM classification version 9.

**Figure 3.**
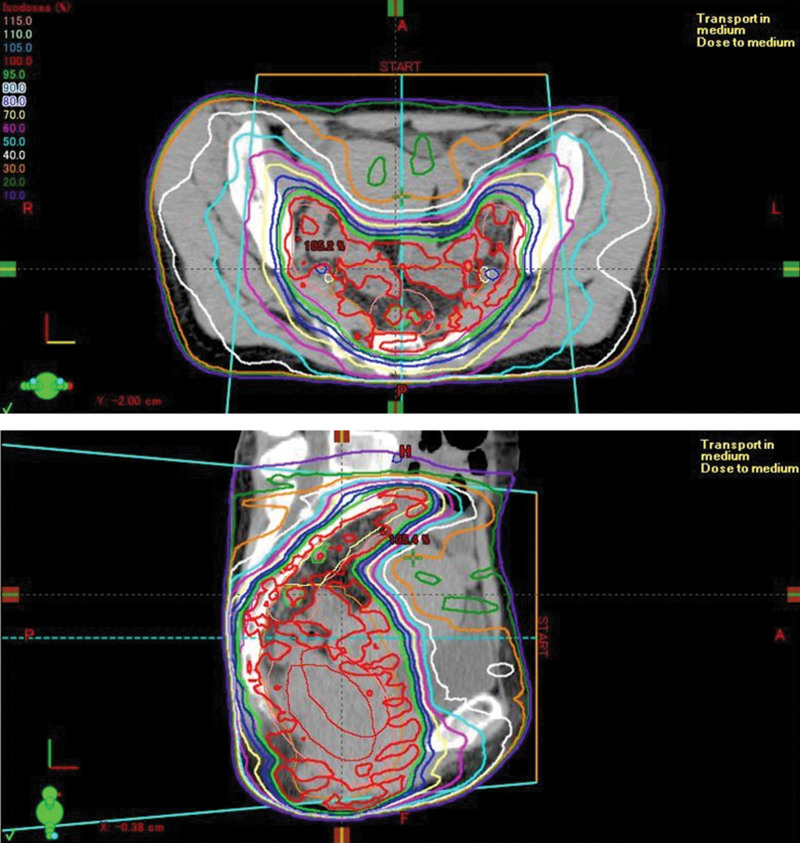
Irradiated areas. Irradiation was planned to include the primary tumor and metastatic lymph nodes in the pararectal and lateral areas. The upper figure shows the transverse section and the lower section presents the sagittal section of the irradiated areas and the dose of intensity-modulated radiation therapy.

The patient had locally advanced colorectal cancer and strongly desired anal preservation. Therefore, preoperative CRT and biweekly S-1 + oxaliplatin + cetuximab were administered simultaneously with intensity-modulated radiation therapy, with a total of 50.4 Gy (1.8 Gy in 28 fractions; Fig. 3). Table 1 summarizes the details of the CRT.

**Table 1:** Chemoradiotherapy schedule (Biweekly SOX + cetuximab).

Drugs	Dosage and usage	Number of trials
S-1	80 mg/m^2^/d, days 1–5 and days 8–12	4 courses
Oxaliplatin	85 mg/m^2^, day 1	
Cetuximab	400 mg/m^2^ (day 1), 250 mg/m^2^(after day 8)	
Radiation	1.8 Gy/fraction, days 1–5 and days 8–12	28 fractions

Following neoadjuvant CRT, the tumor showed a trend toward marked shrinkage, with the rectal wall clearly delineated on MRI (Fig. 2D). All previously enlarged lymph nodes showed a tendency to shrink on MRI and computed tomography (Fig. 2E and F); however, the presence of residual tumor (T3, N0, M0, stage II) was also noted, and the preoperative treatment was determined as a partial response. Laparoscopic partial intersphincteric resection was performed 5 weeks postneoadjuvant CRT.

Histopathological examination of the resected specimens revealed no viable cancer cells in any of the main tumor lesions. Moreover, no obvious lymph node metastases were detected, and the patient was considered to have achieved a pathological complete response (pCR). Moreover, the superior rectal lymph nodes (the central nodes, Fig. 2C and F) outside the irradiated area had also shrunk, resulting in confirming a pCR.

All analyses were conducted in accordance with the Declaration of Helsinki and were approved by the relevant Institutional Ethics Review Board (R04-08). Written informed consent was obtained from the patient for treatment, as well as for publication of this report and any accompanying analyses.

## 3. Discussion

Here we reported a rare case of rectal SCC in which a complete response was successfully achieved with novel CRT comprising oxaliplatin, S-1, cetuximab, and simultaneous radiation.

Adenocarcinomas comprise more than 90% of malignancies affecting the rectum, with the rare occurrence of SCCs. The first case of rSCC was described by Raiford.^[4]^ Primary colorectal SCC is defined when the following 4 criteria are met: presence of pathological features and immunohistochemical profiles of SCC without glandular differentiation; possibility of SCC metastasis or direct invasion of other tissues or organs was excluded; no squamous-lined fistula tract in the affected bowel; absence of tumor extension from the anal squamous epithelium.^[5]^

From 1946 to 2015, based on the Surveillance, Epidemiology, and End Results database of the National Cancer Institute, 142 cases of rSCC were identified by Guerra et al,^[6]^ with the patient age ranging between 39 and 93 years, and the average age at diagnosis being 63 years. Patients were predominantly women, accounting for 57.4% of all cases, with no apparent ethnic or geographic predisposition.^[6]^ Historically, patients with rSCC were managed similarly to rectal adenocarcinomas, primarily relying on low anterior resection or abdominoperineal resection, well-known to be associated with substantial morbidity (13%–46%) and mortality (1%–7%).^[7–9]^ Dyson et al^[10]^ have reported that the prognosis of patients with rSCC who underwent surgical treatment was poor.

Currently, the treatment of rSCC is based on recommendations for anal SCC (aSCC) owing to their common histology. The standard treatment for aSCC involves primary CRT (50.4 Gy of radiotherapy over 28 days) comprising mitomycin C (MMC) and 5-fluorouracil (5-FU).^[11,12]^ Salvage surgery for aSCC can be beneficial in some patients to address recurrent or persistent disease beyond 26 weeks; however, in an increasing number of recent cases, the “wait-and-see” approach has been the treatment of choice.^[13]^

Guerra et al^[6]^ have performed a literature review (487 articles, spanning from 1946 to 2015), revealing that patients with rSCC who underwent definitive CRT achieved a superior overall survival (86%) compared with those who underwent surgery (48%). Likewise, CRT also improved local recurrence (25% vs 10%) and metastasis rates (30% vs 13%) when compared with upfront surgery.^[6]^ Upon analyzing a small cohort, Song et al^[8]^ have concluded that CRT can provide more favorable disease-related outcomes, sphincter preservation, and morbidity profile. Accordingly, Astaras et al^[3]^ have recommended a radiotherapy-based treatment strategy for patients with rSCC. However, given the rarity of rSCC, standard chemotherapy regimens for rSCC are yet to be established. Several reports have documented the use of 5-FU + MMC or cisplatin with radiation therapy for rSCC based on the treatment of aSCC.^[3]^

The use of MMC presents considerable challenges, given the higher hematological toxicity rates in patients who received radiotherapy with 5-FU plus MMC than in those administered 5-FU alone or 5-FU plus cisplatin. MMC is highly toxic and has been discontinued owing to its background.^[14]^

In recent years, the use of oxaliplatin instead of cisplatin has been documented in patients with advanced gastric cancer because cisplatin-based regimens are associated with high toxicity, including nausea, acute kidney injury, and hearing impairment, which can negatively impact the patient’s quality of life.^[15]^ Moreover, renal function is likely to be compromised during treatment with cisplatin-containing regimens. Even with adequate hydration to prevent nephrotoxicity, oxaliplatin has the added benefit of not affecting renal function. Combined regimens of antimetabolites with oxaliplatin have also been employed to treat rSCC.^[16]^ Hence, we used oxaliplatin to develop a treatment regimen for this patient.

S-1 is a novel anticancer agent derived from 5-FU and comprises an orally active combination of tegafur, gimeracil, and oteracil at a molar ratio of 1:0.4:1.^[17]^ Accumulated evidence suggests that S-1 exerts higher antitumor activity, fewer side effects, and excellent biological availability than other FU compounds.^[18,19]^ Funahashi et al^[20]^ have reported the treatment of 3 cases of primary rSCC by administering chemoradiation therapy with S-1. Radiation therapy was delivered at a total dose of 59.4 Gy in 1.8 Gy/fractions over 33 days, and S-1 (80 mg/m^2^/d) was administered orally during radiation therapy. All patients responded completely to treatment. However, 1 patient died of bone and liver metastases 1 year and 2 months postsurgery.^[20]^ Hence, S-1 alone may be insufficient to treat distant metastases from rSCC. Accordingly, we employed oxaliplatin and S-1 combination as the base chemotherapy to treat the patient in this case report.

Considering the treatment of head and neck cancer, cetuximab is reportedly useful in combination with chemoradiation therapy to treat SCC.^[21]^ Although cetuximab with chemoradiation therapy has been reported for rectal adenocarcinoma, there are no reports documenting definitive efficacy.^[22]^ Given that our case involved the treatment of SCC of the rectum, we included cetuximab in this treatment, referring to the treatment of head and neck SCC, ultimately achieving a complete response. In the current case, the tumor had clearly shrunk; moreover, the superior rectal nodes (central nodes, Fig. 2C and F) outside the irradiated area had also shrunk, resulting in a pCR. This suggests that SOX with cetuximab without radiation may be effective for treating metastatic lesions without radiation. The patient had no recurrence at 12 months posttreatment.

The limitations of this report include, as a case report, using 3 types of drugs and radiation, regarding the neoadjuvant setting. A large-scale study might be required for establishing the safety and efficacy of these treatment regimens.

In conclusion, the present treatment elicited substantial tumor shrinkage, and SOX plus cetuximab with radiation may be beneficial not only for locally advanced rSCC but also for addressing metastatic rSCC. Hence, additional cases are warranted to confirm the potential of SOX plus cetuximab with radiation for SCC of the rectum.

## Acknowledgments

The authors thank Dr Tokuhiro Ishihara, Dr Toshiaki Kamei, and Yosuke Nagahiro for their pathological analysis of this work.

## Author contributions

**Data curation:** Yuta Fujise, Shoichi Hazama, Toshiyuki Fujii, Motoshige Inoue, Shotaro Takahashi.

**Investigation:** Yuta Fujise, Shoichi Hazama, Toshiyuki Fujii, Motoshige Inoue.

**Methodology:** Yuta Fujise, Shoichi Hazama, Toshiyuki Fujii, Motoshige Inoue.

**Writing—original draft:** Yuta Fujise, Shoichi Hazama.

**Writing—review & editing:** Yuta Fujise, Shoichi Hazama, Kazuya Yoshida, Akihiko Ikeda, Hiroshi Hashiyada, Kembu Nakamoto, Aogu Yamashita, Keisuke Hino, Kiwamu Okita.

**Conceptualization:** Shoichi Hazama, Keisuke Hino, Kiwamu Okita.
